# Attitudes and Practices of Female University Students in Saudi Arabia Regarding the Cosmetic Use of Careprost (Bimatoprost) Eye Drops

**DOI:** 10.7759/cureus.56233

**Published:** 2024-03-15

**Authors:** Yazeed F Alshahrani, Sami Alghamdi, Abdulmajeed Alkhathami, Abdulmohsen Alshahrani, Sultan M Alshahrani, Abdullah Korkoman

**Affiliations:** 1 Department of Emergency, King Abdullah Hospital, Bisha, SAU; 2 Department of Pharmacy, King Khalid University, Abha, SAU; 3 College of Medicine, University of Bisha, Bisha, SAU

**Keywords:** saudi arabia, practices, attitudes, cosmetic, hypotrichosis, eyelashes, careprost 0.03, bimatoprost eye drops

## Abstract

Background and objective

Bimatoprost ophthalmic solution (0.03%) is used for the treatment of ocular hypertension. However, one of the side effects of this prescription is that it causes overgrowth of eyelashes, causing hypertrichosis. Therefore, the Bimatoprost ophthalmic solution was rebranded to be used for cosmetic purposes. This study aims to assess the awareness and practices of female university students regarding the use of Careprost (Bimatoprost, Latisse, Allergan, Inc., Irvine, CA) for cosmetic purposes.

Methodology

A descriptive cross-sectional study was conducted among female students at Bisha University, including those from medical and non-medical colleges, spanning from November 2022 to February 2023. All participants who completed the study questionnaire were considered for analysis, but those who had missing answers were excluded from the study. The total number of participants was 305, representing an 81.2% response rate out of the 376 surveys distributed.

Results

A total of 305 students completed the survey, with approximately 132 (54.5%) from the medical college and 173 (65.3%) from the non-medical college. Approximately 32 (24.2%) of participants from the medical college and 51 (29.4%) from the non-medical college understood that Bimatoprost drops can be used for the elongation of eyelashes. More than half of the participants were not aware of the side effects of Careprost (0.03%), including 65 (49.2%) medical students and 108 (62.7%) non-medical students. In total, 42 (13.77%) of the participants believed that Careprost (0.03%) could be administered without a prescription. Among the participants, 75 (24.59%) reported that they had previously used Careprost (0.03%) eye drops. Additionally, more than one-fourth of the participants (83, 27.2%) thought that Careprost (0.03%) could be used for eyelash elongation.

Conclusions

This study revealed that female university students had a poor level of awareness and practices about the cosmetic uses of Careprost (0.03%) eye drops for eyelashes. A better awareness level was noted regarding the side effects of Careprost drops, which may have contributed to a low utilization rate among female students.

## Introduction

Bimatoprost is a prostamide F2α analog, including a chemical structure like prostaglandin F2α analogs [1-3]. Prostaglandin receptors are located in the dermal papilla beside the outside root sheath of the hair follicle, which is mainly tangled in the development and regrowth of the hair follicle [1]. Nevertheless, research on animals has shown that the use of Bimatoprost alters the eyelash hair cycle by increasing the percentage of hair follicles in the anagen phase and extending its length [2].

Eyelashes hypotrichosis is a condition featured by an inadequate number of eyelashes [4]. Bimatoprost (0.03% solution, Latisse, Allergan, Inc., Irvine, CA), which is comparable to the ophthalmic solution used to treat glaucoma, has been authorized by the U.S. Food and Drug Administration (FDA) to help individuals with hypotrichosis of the eyelashes grow longer, thicker, and darker lashes [5,6]. Bimatoprost can be used locally, usually with few adverse effects, if it is administered to the base of the lashes near the edge of the lid [7,8]. To stimulate the development of eyelashes, bimatoprost is applied to the lashes and lid margins near the surface of the eye. However, if it is improperly placed on the surface of the eye, adverse effects may result [8].

According to reports, Bimatoprost use in the eyes has been related to increased iris pigmentation. When topical prostaglandin is applied, older patients may experience a greater shift in iris color. Bimatoprost and ocular prostaglandin analogs are linked to conjunctival hyperemia [9,10].

Knowledge and awareness regarding the efficacy and adverse effects associated with Bimatoprost use had a significant role in promoting rational drug use among the population. There is an absolute scarcity of awareness of adverse effects associated with the irrational use of Bimatoprost among adult females. Therefore, this study is the first of its kind in the entire kingdom and would help to identify the awareness level, practices, and factors associated with the use of Bimatoprost among adult females. This would further help in designing interventional measures necessary for creating awareness regarding the safe and rational use of Bimatoprost by adult females.

## Materials and methods

Study design and sample size calculation

A descriptive cross-sectional study was conducted among female students at Bisha University in Bisha, Saudi Arabia, between November 2022 and February 2023. It was distributed to students via a Google Form link, and data collection continued for four months. The population of Bisha University, located in the Aseer region, the southern region of Saudi Arabia, is approximately 17,000. The α-level was set to 5%, and the confidence interval was set to 95% with 5% precision. The number of participants needed for this study was calculated using the following formula:

 n = (Z^2 p(1 - p))/d^2

where n is the sample size, Z is the Z statistic corresponding to the confidence level (commonly chosen as Z = 1.96 for a good estimation of sample size), and p denotes the expected prevalence or proportion Using this equation, the number of subjects that should be included became 376. The total number of participants who volunteered for the survey was 305, yielding a response rate of 81.2% out of the 376 surveys distributed.

Questionnaire

A self-administered, structured, and closed questionnaire was designed based on a previously published study [11] and subsequently modified to suit the female students of Bisha University. A pilot test was conducted to assess the validity of the questionnaire. Four researchers revised the survey scientifically, and a linguistic professional evaluated the study’s language. The Cronbach alpha factor was calculated for 12 pilot study participants and assessed as 0.83. The questionnaire was prepared in English and translated into Arabic by a specialized translator. Female students of Bisha University aged 18 or older (enrolled as university students) and willing to answer questionnaires were included in the study. Participants who were unwilling to provide their consent or were not university female students were excluded. Data on students’ attitudes toward using Careprost (0.03%) w/v eye drops as a cosmetic tool were collected. The online survey included two sections: the first part focused on sociodemographic data, and the second part focused on participants’ attitudes and practice toward the Bimatoprost drops. The questionnaire was written in Google Forms, which required five minutes on average to complete.

Statistical analysis

We evaluated the surveys and cleaned, coded, and entered the data into SPSS Version 20 for statistical analysis (IBM Corp., Armonk, NY). We used both inferential and descriptive statistics to achieve our results. Descriptive analysis based on the frequency and percentage distribution was done for all variables, including participants’ age, academic year, type of college (medical or nonmedical) knowledge items, and participants’ practice and attitude regarding Careprost (0.03%) w/v eye drops. The study questions and characteristics were compared between medical and nonmedical students using an independent Student’s t-test for continuous variables and a chi-square (χ^2^) test for categorical data. *P*-value less than 0.05 was statistically significant.

Ethical considerations

Data collection processes were standardized, and no personal information about the participants was collected or stored. Throughout the investigation and data processing, the remaining materials were kept confidential. Participants' agreement was sought before the start of the survey. They were not requested to provide their ID or any other personal data. The study's participation was entirely voluntary.

The study followed the World Medical Association (WMA) Declaration of Helsinki: Ethical standards for medical research involving human people, as amended by the 59th WMA (ECM#2021-5415), Seoul, Korea, in this study. No personally identifiable information about the participants was collected. In addition, the Research Ethics Committee at King Khalid University (HAPO-06-B-001) reviewed and agreed on this project (approval no. ECM#2021-4101; approval date March 09, 2021).

## Results

Demographic characteristics

A total of 305 students completed the survey. Approximately 132 (54.5%) of the participants were from the medical college, while 173 (65.3%) were from the nonmedical college and aged between 21 and 23 years. Additionally, 37 (28%) of the participants were medical students, and 77 (44.5%) were nonmedical students in their fourth year. Table 1 summarizes the sociodemographic characteristics of the study participants.

**Table 1:** Sociodemographic data of female students of Bisha University, Saudi Arabia.

Sociodemographic data	Medical, *n* (%)	Nonmedical, *n* (%)	*P*-value
Age (years)			
18-20	41 (31%)	41 (23.6%)	0.241
21-23	72 (54.5%)	113 (65.3%)	
24-26	13 (9.8%)	15 (8.6%)	
>26	6 (4.5%)	4 (2.3%)	
Academic year			
First year	20 (15.1%)	18 (10.4%)	0.056
Second year	19 (14.3%)	22 (12.7%)	
Third year	35 (26.5%)	34 (19.6%)	
Fourth year	37 (28%)	77 (44.5%)	
Fifth year	5 (3.7%)	2 (1.1%)	
Sixth year	16 (12.1%)	20 (11.5%)	

Knowledge of participants regarding Careprost (0.03%) eye drops

The majority of the participants, 40 (30.3% of medical students) versus 76 (43.9% of non-medical students), were not aware of what Careprost is used for. The participants who mentioned that Careprost (0.03%) is approved by the FDA were 40 (30.3% of medical ) and 105 (32.3% of medical). In total, 42 (13.77%) of the participants think that Careprost (0.03%) could be administered without a prescription. More than half of the participants, 67 (50.7% medical students) and 88 (50.8% non-medical students), did not know about the precautions associated with Careprost drops. Table 2 shows the knowledge of participants regarding Careprost (0.03%) eye drops.

**Table 2:** Knowledge of participants regarding Careprost (0.03%) eye drops. FDA, U.S. Food and Drug Administration

	Medical	Nonmedical		*P*-value
What is Careprost (0.03%)?				
Used for ocular hypertension	33 (25%)		23 (13.2%)	0.012
Used for elongation of eyelashes	32 (24.2%)		51 (29.4%)	0.13
Used for glaucoma	11 (8.3%)		11 (6.35%)	0.92
All of the above	16 (12.1%)		12 (6.9%)	0.35
Don’t know	40 (30.3%)		76 (43.9%)	0.028
Is Careprost (0.03%) approved by the FDA?				
Yes	40 (30.3%)		57 (32.9%)	0.046
No	19 (14.3%)		11 (6.3%)	0.65
Don’t know	73 (55.3%)		105 (60.6%)	0.063
Which is correct about the uses of Careprost?				
Used with a prescription	47 (35.6%)		57 (33%)	0.21
Used without a prescription	24 (18.1%)		19 (11%)	0.23
Used for cosmetic purposes	18 (13.6%)		25 (14.5%)	0.098
Don’t know	43 (32.5%)		72 (41.8%)	0.16
Which of the following are the side effects of Careprost drops?				
Redness of the eye	29 (21.9%)		29 (16.8%)	0.08
Periorbital pigmentation	27 (20.4%)		21 (12%)	0.06
Iris pigmentation	11 (8.3%)		15 (8.7%)	0.86
Don’t know	65 (49.2%)		108 (62.7%)	0.069
Which of the following is a precaution in Careprost use?				
Allergy to Careprost or other eye drops	40 (30.3%)		49 (28.3%)	0.69
Planned or have a history of eye surgery	16 (12.2%)		18 (10.4%)	0.71
Dry eye	9 (6.8%)		18 (10.4%)	0.12
Don’t know	67 (50.7%)		88 (50.8%)	0.87

The practice of Careprost (0.03%) w/v eye drops among study participants

**Figure 1:**
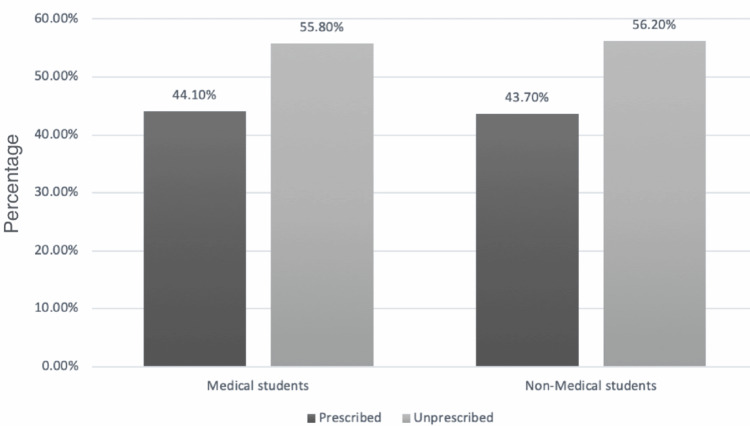
Using Careprost (0.03%) eye drops with and without prescription among medical and nonmedical female university students.

The majority of the participants (81, 67.4%, medical students, and 141, 81.5%, nonmedical students) had never used Careprost drops in their lifetime. Concerning the reason for using Careprost drops, 11 (34.6% of non-medical students) used them for cosmetic purposes, while medical students used them as sterile drops. Table 3 shows the practice of Careprost (0.03%) w/v eye drops among study participants. Regarding the use of Careprost, 24 (56.8% medical students) and 18 (55.8% nonmedical students) had used it without a prescription, as shown in Figure 1.

**Table 3:** Practice of Careprost (0.03%) w/v eye drops among study participants.

Practice items	Medical	Nonmedical	*P*-value
Previously used Careprost (0.03%)			
Yes	43 (32.5%)	32 (18.4%)	0.007
No	89 (67.4%)	141 (81.5%)	0.032
Reason for using Careprost (0.03%)			
Cosmetic for eyelashes	9 (20.9%)	11 (34.6%)	0.33
For glaucoma	7 (16.2%)	5 (15.6%)	0.52
Moisturizing drops	8 (18.6%)	7 (21.8%)	0.87
Eye redness/inflammation	7 (16.2%)	6 (18.7%)	0.63
Sterile drops	12 (27.9%)	3 (9.3%)	0.047
Drops were prescribed/unprescribed			
Prescribed	19 (44.1%)	14 (43.7%)	0.67
Unprescribed	24 (55.8%)	18 (56.2%)	0.32
Previously used Careprost (0.03%) for eyelashes			
Yes	33 (76.7%)	21 (65.6%)	0.21
No	10 (23.2%)	11 (34.3%)	0.37

## Discussion

This study was conducted to evaluate and assess the awareness of female university students regarding the rationale for using Careprost (Bimatoprost) eye drops. Careprost is an eye care medication comprising Bimatoprost as the major element. The active component belongs to the prostaglandin analog category and is used for lowering intraocular pressure in glaucoma patients and treating hypotrichosis of eyelashes [12]. Women often desire longer, fuller, and thicker eyelashes to enhance their appearance. Increased eyelash growth is reported to have a positive psychological impact [13,14]. Recently, the FDA approved a new and simple option for enhanced eyelashes, the topical Bimatoprost ophthalmic solution (0.03%). This solution improves eyelash growth, increasing length, thickness, fullness, and darkness to treat hypotrichosis of the eyelashes [15]. Recent studies on animals reported that using Bimatoprost results in various changes to the hair cycle of eyelashes. A two-week course of Bimtaoprost showed a better result in the proportion of follicles during the anagen phase of the hair cycle. It is understood that Bimatoprost drops promote the conversion from the telogen to anagen phases, resulting in a prolonged duration of anagen. These changes in the length of the anagen phase may likely lead to observable enhancement in the length of eyelashes in association with treatment [16,17].

The results of this study showed that only 56 (18.3%; 25% medical vs. 13.2% nonmedical) of the female students surveyed were knowledgeable regarding Careprost (0.03%) eye drops and their uses, which is higher than a study conducted in Saudi Arabia (13%) [11]. A total of 116 (38%) of the female students in the study, with 30.3% from the medical group and 43.9% from the nonmedical group, were not aware of the uses of Careprost (0.03%) w/v. It was reported that 83 (27.2%) of participants, with 24.2% from medical and 29.4% from non-medical colleges, perceived it as drops for the elongation of eyelashes, which is relatively similar to a study in Saudi Arabia (22.3%) [11]. Additionally, 22 (7.2%) were aware of its use as an ocular hypotensive drug for glaucoma. Furthermore, about one-third (31.8%) of the female students in the study believed that FDA-approved Careprost (0.03%) eye drops are for cosmetic use, while more than half (58%) were unaware of this issue. Being used with a medical prescription was reported by 104 (34.1%) of the study's female students, with 35.6% from the medical group and 33% from the non-medical group. Only 14% thought that it is safe for cosmetic use. More than half of the participants were not aware of the side effects of Careprost (0.03%), with 49.2% from the medical group and 62.7% from the nonmedical group, which is significantly higher than the study conducted in Saudi Arabia (19.7%) [11]. Half of the female university student participants reported that they did not know about the precautions for the use of Careprost (0.03%). Regarding the practice of Careprost (0.03%) w/v eye drops among the female students in the study, about one-fourth of the participants reported using Careprost (0.03%). The most reported indication for using the eye drops was its cosmetic effect on eyelashes, with more than one-fourth of those who used the drops. This percentage was lower than the findings of the study conducted in Saudi Arabia (45%) [11]. Only 49 (16%) of them used the drops for treating glaucoma. Also, more than half of the female students who used the eye drops did so without a prescription from healthcare staff. Also, exactly 220 (72%) of participants, with 76.7% from the medical group and 65.6% from the nonmedical group, had previously used Careprost (0.03%) eye drops for eyelashes. This may be attributed to their poor awareness regarding its cosmetic use and role in enhancing eyelash length and thickness.

Limitations of the study

This study was one of the first in Saudi Arabia to assess the awareness of female university students regarding Careprost (0.03%) eye drops and their cosmetic purposes. This limitation restricted the ability to compare the current study findings with others in the literature and map the study participants' awareness level relative to globally reported awareness. Nevertheless, there are certain drawbacks to this study because, at the time of data collection, community-based data gathering was not practical. Thus, the study relied on a self-reported survey from one university in the nation. In addition, endogenous variables such as weak English skills, medication illiteracy, and limited information about the use of Careprost (0.03%) eye drops may have played a significant role in participants' attitudes. Further research is needed to include more female participants from all parts of the country.

## Conclusions

This study revealed that female university students had a poor level of awareness and practices regarding the cosmetic uses of Careprost (0.03%) eye drops for eyelashes. A better awareness level was observed regarding the side effects of Careprost drops, which may have contributed to a lower utilization rate among female students. Large-scale studies are recommended to precisely assess community awareness and preparedness for using Careprost (0.03%) eye drops and evaluate the associated side effects relative to its benefits. Health education should be provided for women covering all aspects of using Careprost (0.03%) eye drops for cosmetic purposes.
